# HCC screening: assessment of an abbreviated non-contrast MRI protocol

**DOI:** 10.1186/s41747-019-0126-1

**Published:** 2019-12-18

**Authors:** Michael Vinchill Chan, Stephen J. McDonald, Yang-Yi Ong, Katerina Mastrocostas, Edwin Ho, Ya Ruth Huo, Cositha Santhakumar, Alice Unah Lee, Jessica Yang

**Affiliations:** 1 Department of Radiology, Concord Repatriation General Hospital, Sydney, NSW Australia; 2 Concord Repatriation General Hospital Clinical School, Faculty of Medicine, University of Sydney, Sydney, Australia; 3 Bankstown-Campbelltown Hospital, South Western Sydney Clinical School, University of New South Wales, Sydney, Australia; 4 Department of Gastroenterology and Hepatology, Concord Repatriation General Hospital, Sydney, Australia

**Keywords:** Carcinoma (hepatocellular), Diffusion magnetic resonance imaging, Liver cirrhosis, Magnetic resonance imaging, Screening

## Abstract

**Background:**

Hepatocellular carcinoma (HCC) guidelines recommend ultrasound screening in high-risk patients. However, in some patients, ultrasound image quality is suboptimal due to factors such as hepatic steatosis, cirrhosis, and confounding lesions. Our aim was to investigate an abbreviated non-contrast magnetic resonance imaging (aNC-MRI) protocol as a potential alternative screening method.

**Methods:**

A retrospective study was performed using consecutive liver MRI studies performed over 3 years, with set exclusion criteria. The unenhanced T2-weighted, T1-weighted Dixon, and diffusion-weighted sequences were extracted from MRI studies with a known diagnosis. Each anonymised aNC-MRI study was read by three radiologists who stratified each study into either return to 6 monthly screening or investigate with a full contrast-enhanced MRI study.

**Results:**

A total of 188 patients were assessed; 28 of them had 42 malignant lesions, classified as Liver Imaging Reporting and Data System 4, 5, or M. On a per-patient basis, aNC-MRI had a negative predictive value (NPV) of 97% (95% confidence interval [CI] 95–98%), not significantly different in patients with steatosis (99%, 95% CI 93–100%) and no steatosis (97%, 95% CI 94–98%). Per-patient sensitivity and specificity were 85% (95% CI 75–91%) and 93% (95% CI 90–95%).

**Conclusion:**

Our aNC-MRI HCC screening protocol demonstrated high specificity (93%) and NPV (97%), with a sensitivity (85%) comparable to that of ultrasound and gadoxetic acid contrast-enhanced MRI. This screening method was robust to hepatic steatosis and may be considered an alternative in the case of suboptimal ultrasound image quality.

## Key points

- An abbreviated non-contrast magnetic resonance imaging (MRI) protocol to screen for hepatocellular carcinoma has been retrospectively investigated in 188 patients (28 of them with 42 malignancies)
- This protocol demonstrated high specificity (93%) and negative predictive value (97%), with a sensitivity (85%) comparable to that of ultrasound and gadoxetic acid contrast-enhanced MRI
- This screening method was robust to hepatic steatosis and may be considered an alternative to screen high-risk patients in the case of suboptimal ultrasound image quality

## Background

Hepatocellular carcinoma (HCC) is the most common primary malignancy of the liver. Globally, it is the fifth most common cancer and the third most common cause of cancer-related mortality as determined by the World Health Organization [1]. Curative treatments are only available when detected at an early stage, where the 5-year survival is 50–70%. In contrast, patients presenting with advanced HCC are only eligible for palliative treatments and have a poor outcome with a median survival of less than 1 year [2, 3]. Therefore, early detection of HCC is crucial in increasing survival, but currently, only four in ten hepatocellular carcinomas are detected at an early stage [4].

Multiple international practice guidelines recommend screening for HCC [5–13]. All recommend screening with ultrasound (generally 6 monthly) for high-risk groups, including all patients with cirrhosis and some non-cirrhotic patients positive for hepatitis B virus (HBV) infection (Table 1). Cirrhosis is the most significant risk factor for HCC, with 85–95% prevalence amongst HCC patients. As a consequence, in patients with cirrhosis, early detection by screening is crucial [8–10]. Some guidelines suggest alpha-fetoprotein (AFP) or other additional biomarkers as an adjunct to imaging even though the evidence is not so strong for smaller HCC [8, 11, 13, 14] Three guidelines based in the Asia-Pacific region do suggest co-screening with AFP [8]. All guidelines recommend further evaluation with multiphase computed tomography (CT) or magnetic resonance imaging (MRI) for patients with a positive screening test [5, 6, 8–13] (Table 1). The impact of ultrasound with AFP for HCC screening was demonstrated in 2004 by Zhang et al. [15] with a large randomised controlled trial that yielded a significant decrease in mortality in a Chinese population with a high prevalence of HBV.

**Table 1:** Summary of guidelines for hepatocellular carcinoma (HCC) screening protocols

Association/society, abbreviation, year [reference number]	Definition of high-risk population to be screened	Method of surveillance (interval when specified by the guideline)
European Association for the Study of the Liver, EASL, 2018 [9]	• Cirrhosis • F3 hepatic fibrosis • Non-cirrhotic hepatitis B patients with intermediate-high HCC risk (PAGE-B score or higher)	Ultrasound (6 months)
Asian Pacific Association for the Study of the Liver, APASL, 2018 [8]	• Cirrhosis with hepatitis B or C infection, genetic haemochromatosis, primary biliary cirrhosis, alpha-1 antitrypsin deficiency, autoimmune hepatitis. • Non-cirrhotic chronic hepatitis B carrier in patients who are: o Asian female > 50 years o Asian male > 40 years o African > 20 years • Family history of HCC	Ultrasound (6 months)
Combined American Association for the Study of Liver Disease, AASLD, 2018 [10]		Ultrasound + AFP (6 months)
Canadian Association for the Study of Liver Hepatocellular Carcinoma, CASL, 2015 [6]		Ultrasound ± AFP (6 months)
National Comprehensive Cancer Network, NCCN, 2018 [5]		Ultrasound ± AFP (6 months)
Korean Liver Cancer Study Group and the National Cancer Center, KLCSG-NCC, 2014 [13]	• Hepatitis B or C positive • Cirrhosis	Ultrasound + AFP (not stated)
Japanese Society of Hepatology, JSH, 2015 [11]		Ultrasound + AFP and other serological markers (3–6 months depending on risk)
American College of Gastroenterology, ACG, 2014 [12]	• Suggestive of cirrhotic patients but not clearly stated	Ultrasound + AFP

*AFP* Alpha-fetoprotein

Meta-analyses have demonstrated an overall wide pooled per-patient sensitivity of 61–94% for ultrasound only, improved to 69–97% for ultrasound with AFP [2, 4, 16, 17]. In the meta-analysis by Hanna et al. [17], the pooled per-lesion sensitivity of ultrasound only is 59.3% (CI 51.3–67.1%). Only two meta-analyses demonstrated a negative likelihood ratio of 0.50–0.51, suggesting a low diagnostic power to exclude HCC [4, 16].

Although ultrasound has improved in recent years, it has visualisation limitations in patients with obesity, steatosis and advanced fibrosis or cirrhosis [7, 9, 17]. Unfortunately, these factors have all been associated with an increased risk of HCC [18–21]. Hence, HCC screening with ultrasound alone remains even more challenging in those with those risk factors [8, 19, 20]. The worldwide prevalence of non-alcoholic fatty liver disease is approximately 25% and is likely to continue to rise, supporting the need for an alternative screening option in this high-risk group [18, 21].

**Fig. 1:**
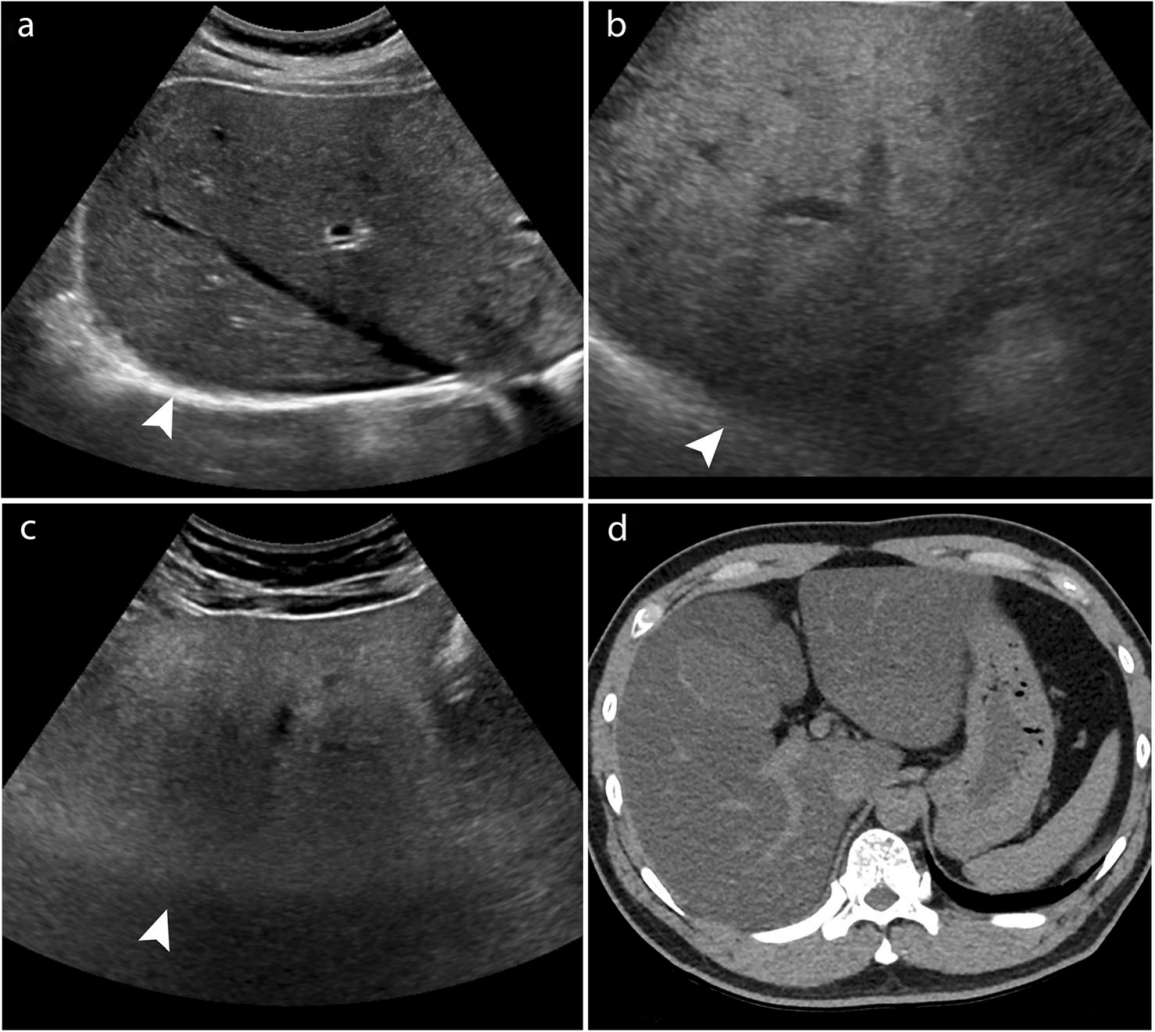
Liver Imaging Reporting and Data System ultrasound visualisation scores. **a** Example of ultrasound visualisation score A: no or minimal limitations with complete visualisation of the diaphragm (arrowhead). **b** Example of ultrasound visualisation score B: moderate limitations. Shadowing and attenuation from heterogeneous liver parenchyma may obscure small masses and less than 50% visualisation of the liver (arrowhead). **c** Example of ultrasound visualisation score C: severe limitations. Marked attenuation in a patient with severe fatty liver leads to poor visualisation of the majority of the liver and diaphragm (arrowhead), with corresponding computed tomography image shown in (**d**)

Limitations in ultrasound lesion visualisation, ranging from minimal (score A) to intermediate (score B) and to severe (score C), are currently addressed in the Liver Imaging Reporting and Data System (LI-RADS) [7] (Fig. 1). Data comparing visualisation score outcomes are lacking [7]. Lower sensitivity in ultrasound screening in patients with non-alcoholic steato-hepatitis when compared to other aetiologies and to cross-sectional imaging has been reported, for instance, by Samoylova et al. [22].

Although multiple meta-analyses demonstrate better sensitivity (per-patient/per-lesion) of contrast-enhanced CT (per-patient 68–70%, per lesion 72–74%) and MRI (81–83% and 79–86%, respectively) [4, 16, 17, 23], they are not cost-effective approaches for a population screening [5, 6, 8–13].

**Fig. 2:**
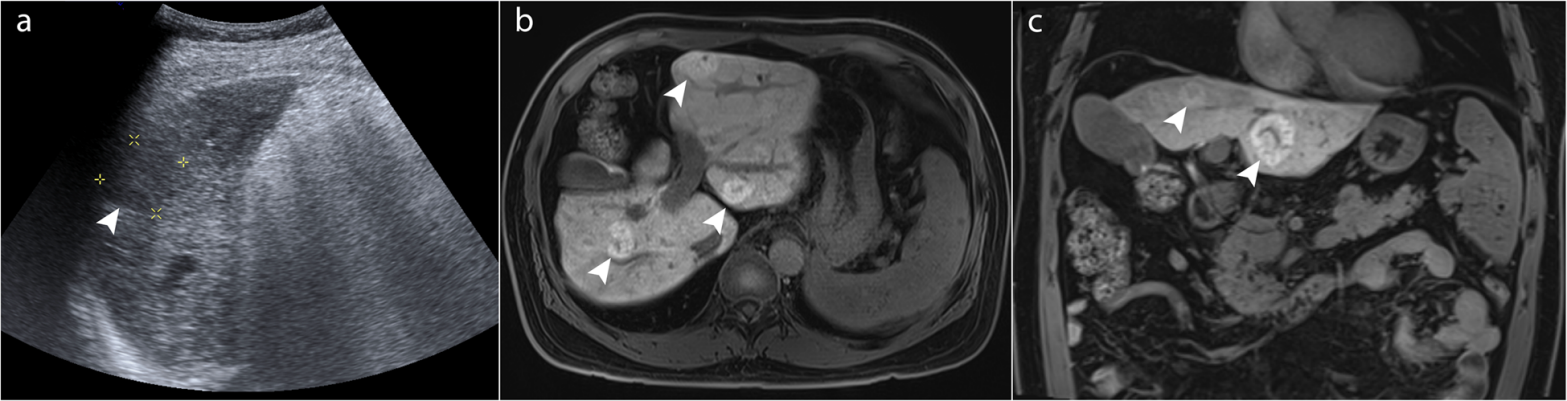
A 59-year-old patient with chronic hepatitis B and cirrhosis who has at least 10 hypoechoic lesions on US (**a**) measuring 2–3 cm (arrowhead). On gadoxetic acid-enhanced magnetic resonance imaging (**b**, **c**), these are shown to be focal nodular hyperplasia (FNH)-like nodules in cirrhosis (arrowheads). This patient cannot be reliably screened with ultrasound. In fact, FNH-like nodules are identical to classic FNH and are benign. They occur in cirrhosis and are believed to originate from acquired hyperplastic responses to vascular alterations associated with cirrhosis

**Fig. 3:**
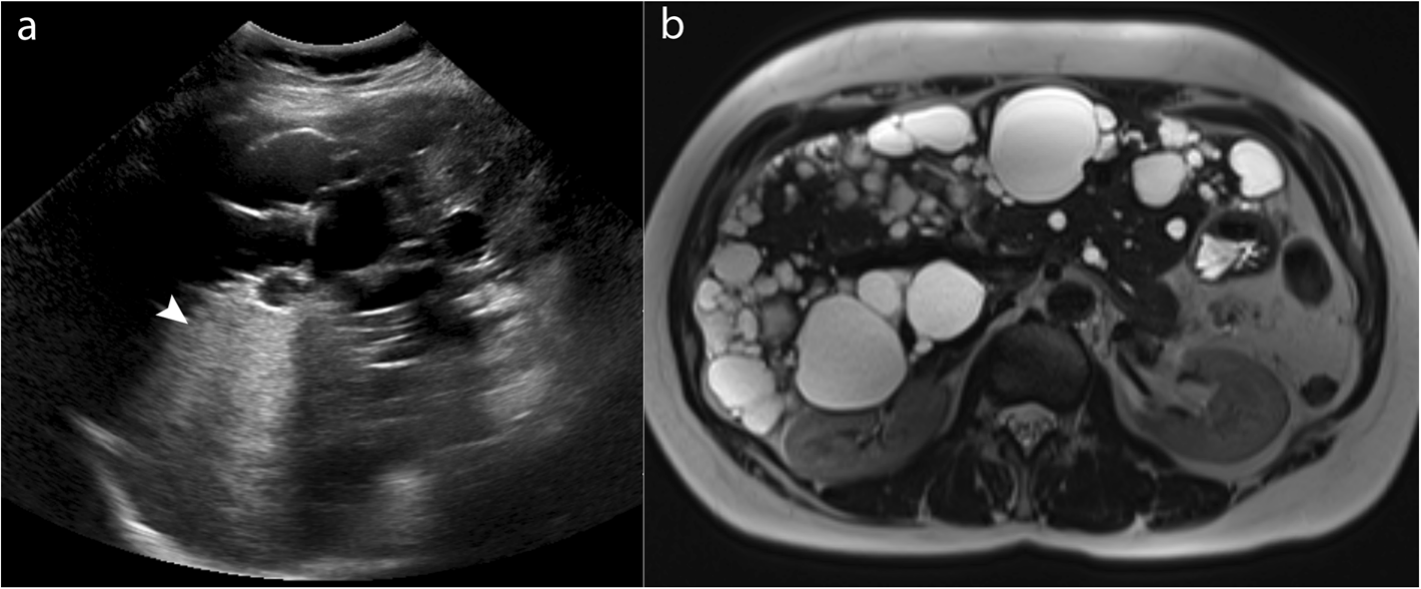
A 58-year-old female patient with chronic hepatitis B and polycystic liver. **a** Heterogeneous posterior acoustic enhancement (arrowhead) limits lesion detection on ultrasound. **b** The cysts do not affect liver parenchymal visibility with MRI

Furthermore, the presence of multiple benign or indeterminate liver lesions such as haemangiomas, regenerative, and dysplastic nodules in cirrhotic patients can confound longitudinal comparison of lesions as well as the detection of new lesions (Figs. 2 and 3). The quality of screening ultrasound is highly operator-dependent, further limiting its use with confidence. Guidelines on how best to screen these high-risk patients who have a suboptimal ultrasound performance are not available [7]. In these patients, clinicians may resort to regular or alternate contrast-enhanced CT or MRI, at regular or increased screening interval or continue with 6 monthly screening with suboptimal ultrasound.

In addition, access to MRI is limited and expensive in many countries. A full contrast-enhanced MRI liver study usually takes 40 min to acquire. In some countries, hepatocyte-specific contrast agent such as gadoxetic acid (Primovist® or Eovist®, Bayer, Leverkusen, Germany) may be difficult to access [8]. Hence, an MRI screening protocol without contrast administration for high-risk patients would be a practical screening tool for those that have suboptimal or non-diagnostic ultrasound. Non-contrast MRI is more accessible, with faster scanning time and lower risk of complications due to cannulation, contrast reactions and gadolinium accumulation [24, 25].

Several abbreviated MRI HCC screening protocols have been developed. Besa et al. [26, 27] utilised an abbreviated methodology with contrast MRI. The former study showed a 80.6% sensitivity and a negative predictive value (NPV) of 90%. Non-contrast MRI sequences mainly based upon diffusion-weighted imaging (DWI) have demonstrated a 48–86% sensitivity and a 85–92% NPV [26, 28].

Our aim was to retrospectively estimate the diagnostic performance of an abbreviated non-contrast MRI (aNC-MRI) protocol to screen high-risk patients including axial T2-weighted, T1-weighted, and DWI sequences.

## Methods

### Subjects

This was a single centre retrospective observational study. Ethics approval by the Sydney Local Health District Human Right and Ethics Committee as a low-negligible risk was obtained. Comprehensive contrast-enhanced liver MRI studies from a single institution at Concord Repatriation General Hospital were identified using a search of the picture archiving and communication system database.

A total of 901 consecutive liver MRI studies from November 2015 to August 2018 were screened for inclusion in the study. All non-contrast MRI studies were eliminated. If a patient had multiple studies, only one study was considered for inclusion. A total of 302 patients with contrast-enhanced liver MRI studies were considered. Studies were excluded if they were after HCC treatment or specifically performed for assessment of liver metastases from a known primary malignancy. Studies of known benign liver lesions such as hepatic adenoma and focal nodular hyperplasia (FNH) and studies showing hepatic infection such as abscesses or primarily biliary pathologies such as primary sclerosing cholangitis were excluded. Studies with excessive artefact (*n* = 7) or missing sequences (*n* = 21) were also excluded. A total of 188 studies of 188 patients met inclusion criteria and entered the analysis. Demographic data, evidence of cirrhosis, HBV/hepatitis C virus status, or other HCC risk factors were recorded for every patient.

### MRI acquisition

The studies were performed on a 3-T MRI unit (Skyra, Siemens, Munich, Germany) with a routine protocol including the following sequences: coronal and axial T2-weighted (echo time 80 and 160 ms), axial fat-saturated T2-weighted, axial T1-weighted Dixon (in-phase, opposed-phase, water-weighted and fat-weighted images), DWI, unenhanced and contrast-enhanced multiphase coronal, and axial T1-weighted sequences. A 30-channel radio-frequency body coil was used. From the routine protocol, the aNC-MRI study was created by selecting the axial T2-weighted sequence with 160-ms echo time, all the four axial T1-weighted Dixon sequences, and the DWI sequences with the apparent diffusion coefficient (ADC) maps. The sequences of the aNC-MRI protocol were anonymised and exported for analysis on a separate viewer. Detailed technical parameters of the aNC-MRI protocol are reported in Additional file 1: Table S1.

### Image analysis

Each study finding was categorised as normal, benign or malignant based on the routine MRI study and report reviewed by the senior investigator (J.Y.). For each scan with malignant findings, the size, liver segment, and LI-RADS category were recorded for each lesion. Studies with more than three malignant lesions were considered to be multifocal. For all studies, the severity of hepatic steatosis was categorised as none, mild, moderate, or severe based on the percentage signal loss between the T1-W Dixon in-phase and opposed-phase sequences with thresholds of 5%, 25%, and 40% [29, 30]. The presence of cirrhosis was determined based on a combination of imaging features, the hepatologist’s imaging request, and the patient’s medical records. Imaging features used to determine cirrhosis include morphology of liver lobes, liver contour, nodules, varices, and ascites [31]. It was possible for patients to have both cirrhosis and steatosis. This was assessed separately by one of the investigators to ensure observation consistency.

The anonymised aNC-MRI studies were loaded onto a separate, independent viewer. Three readers – two abdominal fellowship trained radiologists (R1 and R3) and one final-year resident (R2) – reviewed all images independently. Each reader was asked to categorise each scan as ‘return to screening’ or ‘needs further imaging’. Within the ‘return to 6-monthly screening’ category, there were subcategories of ‘normal findings’ or ‘benign finding(s)’. Within the ‘needs further imaging’ category, there were subcategories of ‘indeterminate’ or ‘malignant’ requiring further input by the reader to assess the size, location and possible other comments for each lesion.

### Statistical analysis

Comprehensive contrast-enhanced MRI was considered the reference standard with respect to the presence or absence of malignant liver lesions. The results from each of the readers were analysed and compared to the categories (normal, benign, or malignant) based on the routine MRI study and report reviewed by the senior investigator on a per-scan and per-lesion basis. Sensitivity, specificity, positive predictive value (PPV), and negative predictive value (NPV) were calculated based on whether the reader had categorised the study as ‘needs further imaging’ or ‘return to 6-monthly screening’, which was the primary endpoint; 95% confidence intervals (CI) were calculated according to the binomial distribution.

Summary receiver operating characteristic (SROC) curves were generated and the area under the curves calculated from the pooled data from the three blinded reviewers using Windows Meta-Disc (Hospital Universitario Ramón y Cajal, Madrid, Spain). Interobserver variability was calculated using Cohen κ statistics with SPSS version 19.0 Mac (IBM Corporation, Armonk, USA). 95% confidence intervals were calculated according to the efficient-score method (corrected for continuity) described by Newcombe, based on the procedure outlined by Wilson. For the comparison of sensitivities, the *p* value was obtained from the confidence interval outlined by Altman and Bland [32–34].

## Results

**Fig. 4:**
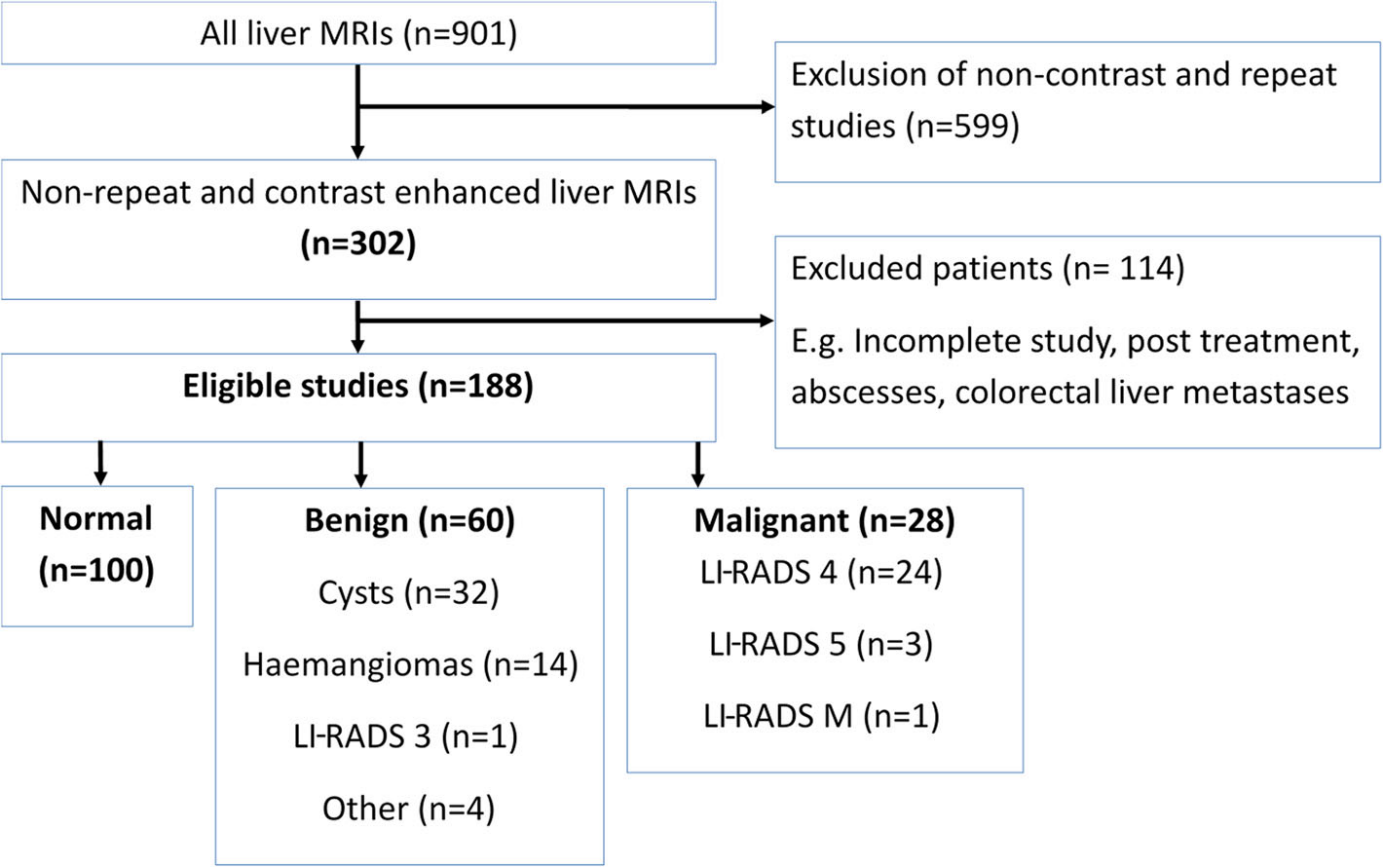
Flow chart of study population

Of the 302 patients with contrast-enhanced liver MRI studies, 188 patients/studies were eligible to be included (Fig. 4), 95 females and 93 males. The patient age ranged from 22 to 89 years (mean 63 years, standard deviation ± 13, range 22–89 years). The clinical characteristics and aetiology of the liver disease of patients are summarised in Table 2. Hepatitis B was the most common cause of liver disease (28 patients, 14.9%). Cirrhosis was present in 44 patients (23.4%), steatosis in 36 (19.1%), and both cirrhosis and steatosis in 4 (2.1%). One hundred patients had no lesions, 60 patients had benign lesions and 28 patients had malignant lesions. In these 28 patients, there was a total of 42 discrete malignant lesions: 31 were LI-RADS 5, 10 LI-RADS 4 and 1 LI-RADS-M.

**Table 2:** Patient demographics and clinical characteristics (*n* = 188)

Sex	95 females, 93 males
Age*	63 ± 13 (22–89)
Cirrhosis	44 (23.4%)
Liver disease aetiology	
HBV	28 (14.9%)
HCV	13 (6.9%)
NASH/NAFLD	9 (4.8%)
Alcohol	7 (3.7%)
Idiopathic cirrhosis	5 (2.7%)
Autoimmune hepatitis	2 (1.0%)
Other	18 (9.6%)
None	106 (5.4%)
Fatty liver	
None	152 (80.9%)
Mild	22 (11.7%)
Moderate	9 (4.7%)
Severe	5 (2.7%)

*HBV* Hepatitis B virus, *HCV* Hepatitis C virus, *NAFLD* Non-alcoholic fatty liver disease, *NASH* Non-alcoholic steatohepatitis

*Mean ± standard deviation (range)

### Per-patient analysis

#### Sensitivity

The aNC-MRI protocol showed a pooled sensitivity of 84.5% for LI-RADS 4, 5, and M (95% CI 74.6–91.2%) (Table 3). The SROC curve had an area under the curve of 0.97. The highest pooled sensitivity was seen in patients with steatosis with a sensitivity of 88.9% (95% CI 74.6–91.2%), followed closely by patients with liver cirrhosis at 86.4% (95% CI 71.5–91.4%). In patients without steatosis or cirrhosis, the pooled sensitivity for all readers was 84.0% (95% CI 73.3–91.1%) and respectively. Overall sensitivity was 75.0 (95% CI 54.8–88.6%) for R1, 82.1 (95% CI 62.4–93.2%) for R2 and 96.4 (95% CI 79.7–99.8%) for R3.

**Table 3:** Per-patient sensitivity, specificity, PPV, and NPV for aNC-MRI for LI-RADS 4, 5, and M categories at full protocol including contrast-enhanced sequences

	Pooled	Reader 1	Reader 2	Reader 3
Overall				
PPV	71/106, 67.0% (57.1–75.6%)	21/30, 70.0% (50.4–84.6%)	23/32, 71.9% (53.0–85.6%)	27/44, 61.4% (45.5–75.3%)
NPV	445/458, 97.2%, (95.1–98.4%)	151/158, 95.6% (90.7–98.0%)	151/156, 96.8% (92.3–98.8%)	143/144, 99.3% (95.6–100.0%)
Sensitivity	71/84, 84.5%, (74.6–91.2%)	21/28, 75.0% (54.8–88.6%)	23/28, 82.1% (62.4–93.2%)	27/28, 96.4% (79.7–99.8%)
Specificity	445/480, 92.7%, (89.9–94.8%)	151/160, 94.4% (89.2–97.2%)	151/160, 94.4% (89.3–97.2%)	143/160, 89.4% (83.3–93.5%)
Cirrhosis present				
PPV	51/61, 83.6% (71.5–91.4%)	14/15, 93.3% (66.0–99.6%)	17/20, 85.0% (61.1–96.0%)	20/26, 76.9% (55.9–90.2%)
NPV	63/71, 88.7% (78.4–94.7%)	23/29, 79.3% (59.7–91.2%)	21/23, 91.3% (70.4–98.4%)	19/19, 100% (79.1–100%)
Sensitivity	51/59, 86.4% (74.4–93.6%)	14/20, 70.0% (45.7–87.1%)	17/19, 89.4% (65.5–98.1%)	20/20, 100% (79.9–100%)
Specificity	63/73, 86.3% (75.8–92.9%)	23/24, 95.8% (76.9–99.8%)	21/24, 87.5% (66.5–96.7%)	19/25, 76% (54.5–89.8%)
No cirrhosis				
PPV	21/47, 44.7% (30.5–59.8%)	8/16, 50.0% (25.5–74.4%)	6/12, 50.0% (22.3–77.7%)	7/19, 36.8% (17.2–61.4%)
NPV	382/386, 99.0% (97.1–99.7%)	128/129, 99.2% (95.1–100%)	130/132, 98.5% (94.1–99.7%)	124/125, 99.2% (95.0–100%)
Sensitivity	21/25, 84.0% (63.1–94.7%)	8/9, 88.9% (50.7–99.4%)	6/8, 75.0% (35.6–95.5%)	7/8, 87.5% (46.7–99.3%)
Specificity	382/408, 93.6% (90.7–95.7%)	128/136, 94.1% (88.3–97.2%)	130/136, 95.6% (90.2–98.1%)	124/136, 91.2% (84.8–95.2%)
Steatosis present				
PPV	8/19, 42.1% (21.1–66.0%)	3/7, 42.9% (11.8–79.8%)	2/3, 66.7% (12.5–98.2%)	3/9, 33.3% (9.0–69.1%)
NPV	88/89, 98.9% (93.0–99.9%)	29/29, 100% (85.4–100%)	32/33, 97.0% (82.5–99.9%)	27/27, 100% (84.5–100%)
Sensitivity	8/9, 88.9% (50.7–99.4%)	3/3, 100% (32.0–100%)	2/3, 66.7% (12.5–98.2%)	3/3, 100% (31.0–100%)
Specificity	22/99, 88.9% (80.6–94.1%)	29/33, 87.9% (70.9–96.0%)	32/33, 97.0% (82.5–99.9%)	27/33, 81.8% (63.9–92.4%)
No steatosis				
PPV	63/87, 72.4% (61.6–81.1%)	18/23, 78.3% (55.8–91.7%)	21/29, 72.4% (52.5–86.6%)	24/35, 68.6% (50.6–82.6%)
NPV	357/369, 96.7% (94.2–98.2%)	122/129, 94.6% (88.7–97.6%)	119–123, 96.7% (91.4–99.0%)	116/117, 99.1% (94.6–100%)
Sensitivity	63/75, 84.0% (73.3–91.1%)	18/25, 72.0% (50.4–87.1%)	21/25, 84.0% (63.1–94.7%)	24/25, 96.0% (77.7–99.8%)
Specificity	357/381, 93.7% (90.6–95.8%)	122/127, 96.0% (90.6–98.5%)	119/127, 93.7% (87.6–97.0%)	116/127, 91.3% (84.7–95.4%)

Data are expressed as ratio, point estimate and, in parentheses, 95% confidence interval

*aNC-MRI* Abbreviated non-contrast magnetic resonance imaging, *LI-RADS* Liver Imaging Reporting and Data System, *PPV* Positive predictive value, *NPV* Negative predictive value

#### Specificity

The aNC-MRI protocol showed a pooled specificity of 92.7% for LI-RADS 4, 5, and M (95% CI 89.9–94.8%) (Table 3). The highest pooled specificity was seen in patients with no cirrhosis and no steatosis, which were 93.6% (95% CI 90.7–95.7%) and 93.7% (95% CI 90.6–95.8%), respectively. Patients with steatosis had a specificity of 88.9% (95% CI 80.6–94.1%) and those with cirrhosis had a specificity of 86.3% (95% CI 75.2–92.9%). The overall specificity was 94.4% (95% CI 89.2–97.2%) for R1, 94.4% (95%CI 89.3–97.2%) for R2, and 89.4% (95% CI 83.3–93.5%) for R3.

#### Negative predictive value

The aNC-MRI showed a pooled NPV of 97.1% for LI-RADS 4, 5, and M (95% CI 95.1–98.4%) (Table 3). The highest NPV were in patients with no cirrhosis at 99.0% (95% CI 97.1–99.7%), followed by those with steatosis at 98.9% (95% CI 93.0–99.9%). The NPV for patients with no steatosis was 96.7% (95% CI 94.2–98.2%), for patients with cirrhosis was 88.7% (95% CI 78.4–94.7%). The overall NPV was 95.6% (95% CI 90.7–98.0) for R1, 96.8% (95% CI 92.3–98.8%) for R2, and 99.3% (95% CI 95.6–100%) for R3.

#### Positive predictive value

The aNC-MRI had a pooled PPV of 67% for LI-RADS 4, 5, and M (95% CI 57.1–75.6%) (Table 3). The highest PPV was in patients with cirrhosis at 83.6% (95% C1 71.5–91.4%), followed by those with no steatosis at 72.4% (95% CI 55.8–91.7%). The PPV for patients with no cirrhosis was 44.7% (95% CI 30.5–59.8%), for patients with steatosis was 42.1% (95% CI 21.1–66.0%). The overall PPV was 70% (95% CI 50.4–84.6%) for R1, 71.9% (95% CI 53.0–85.6%) for R2, and 61.4% (95% CI 45.5–75.3%) for R3.

### Per-lesion analysis

On a per lesion basis, the overall pooled sensitivity for LI-RADS 4, 5, and M was 77% (95% CI 68.5–83.8%). For readers 1, 2, and 3, the sensitivity was 69.0% (95% CI 52.8–81.9%), 66.7% (95% CI 50.4–80.0%), and 95.2% (95% CI 82.6–99.2%), respectively. For lesions being 20 mm or larger in diameter, the pooled sensitivity was 85.3% (95% CI 74.8–92.1%). For lesions being less than 20 mm in size, the pooled sensitivity was 64.7% (95% CI 50.0–77.2%). All three readers had a higher sensitivity for lesions 20 mm or larger compared to lesions smaller than 20 mm in diameter, but this was not significant (*p* > 0.05) (Table 4).

**Table 4:** Per-lesion sensitivity for aNC-MRI for LI-RADS 4, 5 and M categories full protocol including contrast-enhanced sequences

	Per-lesion sensitivity
Pooled overall	
All malignant lesions (*n* = 42)	97/126, 77.0% (68.5–83.8%)
< 20 mm	33/51, 64.7% (50.0–77.2%)
≥ 20 mm	64/75, 85.3% (74.8–92.1%)
Reader 1	
All malignant lesions	29/42, 69.0% (52.8–81.9%)
< 20 mm	10/17, 58.9% (33.5–80.6%)
≥ 20 mm	19/25, 76.0% (54.5–89.8%)
Reader 2	
All malignant lesions	28/42, 66.7% (50.4–80.0%)
< 20 mm	7/17, 41.2% (19.4–66.5%)
≥ 20 mm	21/25, 84.0% (63.1–94.7%)
Reader 3	
All malignant lesions	40/42, 95.2% (82.6–99.2%)
< 20 mm	16/17, 94.1% (68.2–99.7%)
≥ 20 mm	24/25, 96.0% (77.7–99.8%)

Data are expressed as ratio, point estimate, and, in parentheses, 95% confidence interval. *aNC-MRI* Abbreviated non-contrast magnetic resonance imaging, *LI-RADS* Liver Imaging Reporting and Data System

### Interobserver variability

The overall interobserver variability measured using Cohen’s κ ranged from 0.51 to 0.57; there was moderate agreement between the readers in patients who required further contrast-enhanced assessment and those who did not (Table 5). The variability remained relatively constant in patients with no fatty liver (κ = 0.54–0.58) and those with cirrhosis (κ = 0.48–0.60). In patients with fatty liver, R1 and R3 demonstrated an agreement (κ = 0.68) higher than that between R1 and R2 (κ = 0.32), as well as between R2 and R3 (κ = 0.24).

**Table 5:** Interobserver variability of aNC-MRI protocol (κ value)

	Readers (κ value)		
	R1, R2	R1, R3	R2, R3
All patients (*n* = 188)	0.54	0.57	0.51
Fatty liver			
Yes (*n* = 36)	0.32	0.68	0.24
No (*n* = 152)	0.58	0.54	0.57
Cirrhosis			
Yes (*n* = 44)	0.49	0.48	0.60
No (*n* = 144)	0.47	0.53	0.25
LI-RADS 4, 5 and M lesions (*n* = 51)	0.23	0.32	0.25
< 20 mm	-0.04	0.30	0.12
≥ 20 mm	0.26	0.23	0.36

*aNC-MRI* Abbreviated non-contrast magnetic resonance imaging, *LI-RADS* Liver Imaging Reporting and Data System

In terms of LI-RADS 4, 5, and M lesions, there was poor to fair agreement between the LI-RADS categories (κ = 0.23–0.32) and with lesions showing a diameter of 20 mm or larger. In lesions smaller than 20 mm, there was poor agreement between R1 and R2 (κ = -0.04), poor to fair agreement between R1 and R3 (κ = 0.30) and poor agreement between R2 and R3 (κ = 0.12).

### Lesions detection according to size and LI-RADS category

**Fig. 5:**
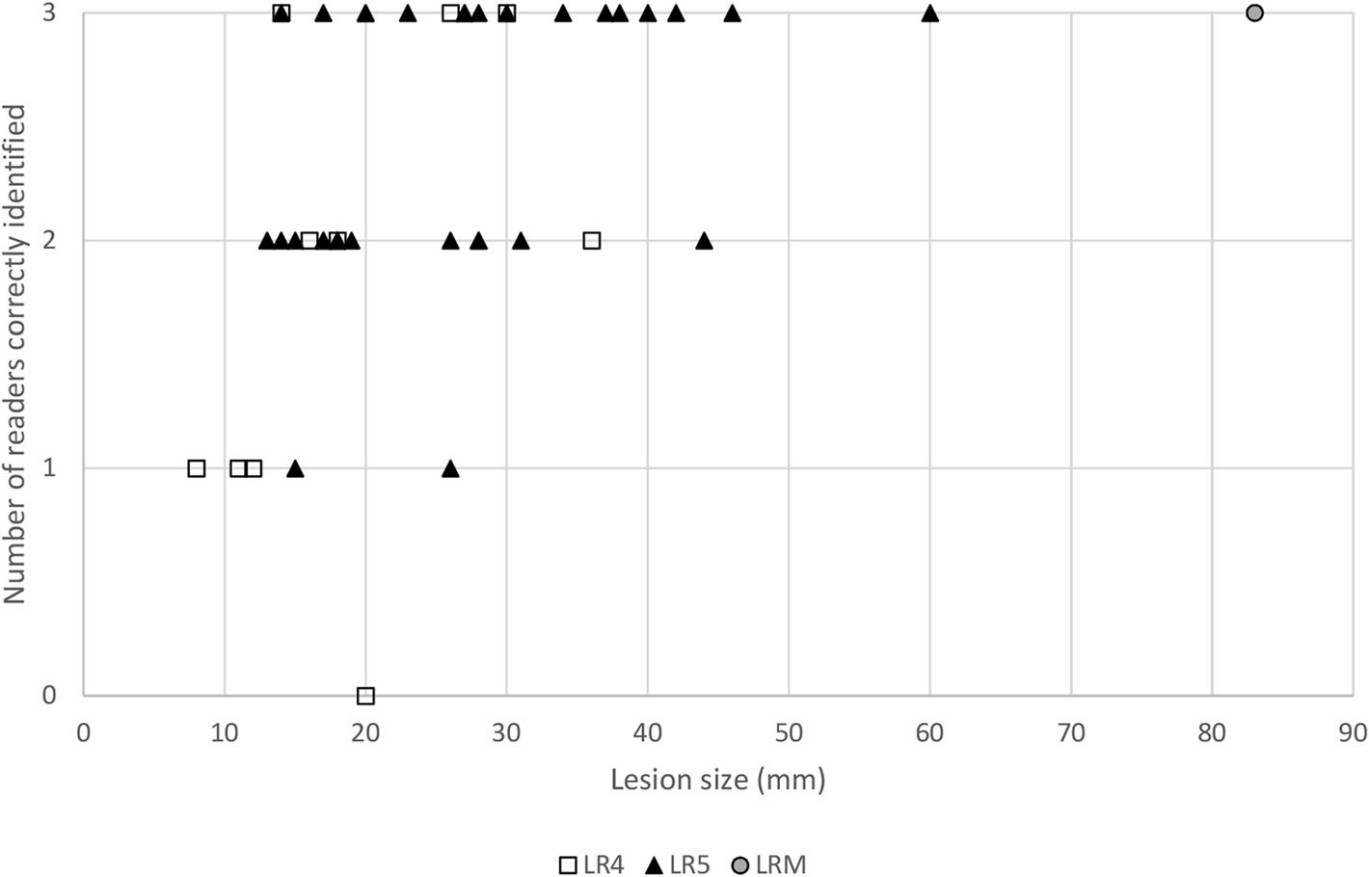
Lesion size and Liver Imaging Reporting and Data System (LI-RADS) category *versus* detection by readers. *LR* LI-RADS

Of the 42 LI-RADS 4, 5, and M lesions, only one was not detected by any of the three readers. This patient also had two other LI-RADS 5 lesions which were detected by two of the readers. Overall, five lesions were detected by one of three readers; and 15 lesions were detected by two of the three readers. Twenty-two lesions were missed by at least one reader, 13 of them (59%) being smaller than 20 mm. Figure 5 illustrates the association between lesion size and lesion detection stratified for LI-RADS 4, 5, and M lesions.

## Discussion

Biannual screening for HCC is critical for the early detection in high-risk patients. It is currently recommended by internationally recognised guidelines as a standard practice. The most recently updated guidelines demonstrate increasing convergence of accepted risk factors to be considered for screening [5, 6, 8–13]. However, surveillance using ultrasound presents a number of limitations in high-risk patients with suboptimal or non-diagnostic scans. These include those with obesity, hepatic steatosis, cirrhosis and multiple benign liver lesions [22]. Currently, no guidelines address screening practices for these patients when ultrasound is inadequate.

In this retrospective study, we have demonstrated that an aNC-MRI protocol could be a potential alternative screening tool. It showed a pooled sensitivity of 84.5% and a NPV of 97.1%. These results are similar to those obtained by studies utilising DWI only [26, 28] and those with an abbreviated contrast-enhanced MRI protocol [26, 28, 35].

There was little difference in sensitivity and NPV between assessment with and without cirrhosis or hepatic steatosis. Our pooled per-patient sensitivities of 86.4% in cirrhotic patients and 88.9% in patients with hepatic steatosis were higher than per-lesion sensitivity of ultrasound as per the meta-analyses by Hanna et al. [17], which was 59.3%. Our NPV of 88.7% in cirrhotic patients and 98.9% in patients with hepatic steatosis could allow us to exclude malignancy, especially in patients with severe hepatic steatosis who otherwise would have a non-diagnostic ultrasound examination. Of the 42 LI-RADS 4, 5, and M lesions, only one was not detected by any of the three readers. The lesion was a 20-mm LI-RADS 4 lesion in segment 8 near the diaphragm. This region in the liver can be difficult to interpret on MRI due to the proximity to the diaphragm and patient respiratory motion. Coincidentally, this is also a region that is often difficult to visualise on ultrasound due to its high position and often can only be seen through intercostal scanning. Detailed assessment of negative likelihood ratios in future meta-analyses of different modalities would be useful in considering the role of combining modalities for future screening studies as shown by the meta-analysis by Colli et al. [16].

We observed a reduced sensitivity for lesions < 20 mm (64.7%) *versus* ≥ 20 mm (85.3%). This compares favourably with the pooled sensitivity of ultrasound (47%) for detecting early stage HCC according to the Milan criteria [4, 36]. Furthermore, blinding our readers from prior studies was not entirely representative of clinical practice and represents a worst-case scenario. When aNC-MRI screening is to be used in the clinical setting, we propose that the patient has an initial baseline contrast-enhanced liver MRI, followed by six monthly serial screening aNC-MRIs used for comparison. This should increase reader confidence, and we expect that it will result in improved sensitivity and specificity.

There is some variability in the appearance of HCC on DWI sequences, but, despite this, it remains a key sequence of unenhanced liver imaging [26, 28, 37]. Whilst DWI is neither extremely sensitive nor specific for HCC [38, 39], it is sensitive for malignancy and remains robust in the setting of hepatic steatosis. In our study, If restricted diffusion is present or suspected in the liver, the screening scan will be considered for further contrast-enhanced assessment, after evaluation in combination with the T1-weighted and T2-weighted sequences (to exclude definite benign lesions such as cyst and haemangioma). Future research on unenhanced liver MRI screening should include studies with larger cohorts, possibly in combination with AFP, and head-to-head analysis *versus* ultrasound such as the prospective randomised MIRACLE-HCC study proposed by An et al. [40].

The main issues with MRI are cost, time and accessibility. Although contrast-enhanced CT is more accessible than contrast-enhanced MRI and both have improved per-lesion sensitivity compared to ultrasound, it is not recommended by any of the screening guidelines [2, 4–6, 8–13, 16, 17]. The economic rationale for abbreviated protocols for screening has been raised by Besa et al. [26] for both unenhanced and contrast-enhanced abbreviated MRI protocols with acceptable sensitivity and NPV in populations with a 2% and 8% HCC prevalence. Of note, the scan time of our aNC-MRI is only one-third of that of our standard liver MRI protocol.

Economic analysis of those patients with LI-RADS US visualisation score C [7] and their outcomes with either ultrasound or MRI with economic analysis may also be useful. We note that a large tertiary or multi-centre institute may be required to generate statistically significant data as only a small proportion of patients screened at our institution fall into this category.

The results of this study must be considered within the context of its inherent limitations. There is potential for bias due to the retrospective study design, performed within a single centre and with a single 3-T MRI, which may not necessarily translate to scanners of different model or field strength. Although this study has a relatively small sample size, it is comparable in size to similar studies that utilised a non-contrast-enhanced series for evaluation [26, 28, 39]. The scans included in our aNC-MRI series were sourced from patients who have had contrast-enhanced MRI performed for any reason (with subsequent criteria for exclusion from the study). However, this still introduces an inherent bias, mainly as not all of the patients would fit the high-risk screening criteria. For clarification, all 28 patients who had a positive MRI screening study satisfied the high-risk criteria, while not all of the patients who had a normal/benign MRI screening study met the high-risk criteria. For the purpose of this study, we felt that the latter would have a minimal adverse effet on the reader's reading outcome. There was significant variability amongst readers, although there was moderate agreement between the readers in patients who required further contrast-enhanced assessment and those who did not. Ideally, more readers of an appropriate level could be utilised to compensate for a relatively small cohort. Finally, histopathological correlation and ultrasound correlation was not available for all MRI studies and missed HCCs on the routine contrast-enhanced assessment cannot be excluded.

In conclusion, this retrospective study of aNC-MRI HCC screening protocol demonstrated an acceptable sensitivity (84.5%) and a high NPV (97.1%), potentially offering an alternative screening tool for high-risk patients who otherwise have a suboptimal screening ultrasound.

## Supplementary information

**Additional file 1:** **Table S1.** MRI protocol for aNC-MRI

## Data Availability

The datasets used and/or analysed during the current study are available from the corresponding author on reasonable request.

## Abbreviations

ADC: Apparent diffusion coefficient
AFP: Alpha-fetoprotein
aNC-MRI: Abbreviated non-contrast magnetic resonance imaging
CI: Confidence interval
CT: Computed tomography
DWI: Diffusion-weighted imaging
FNH: Focal nodular hyperplasia
HBV: Hepatitis B virus
HCC: Hepatocellular carcinoma
LI-RADS: Liver Imaging Reporting and Data System
MRI: Magnetic resonance imaging
NPV: Negative predictive value
PPV: Positive predictive value
SROC: Summary receiver operating characteristic
