# Supplementary material for: HCC screening: assessment of an abbreviated non-contrast MRI protocol
Source: Eur Radiol Exp. 2019 Dec 18;3:49. doi: 10.1186/s41747-019-0126-1 (PMC6920271; doi:10.1186/s41747-019-0126-1)
Supplement: Supplementary file 1 — Additional file 1: Table S1. MRI protocol for aNC-MRI [file 41747_2019_126_MOESM1_ESM.docx]

Additional file 1: **Table S1.** MRI protocol for aNC-MRI

| Sequence | T2 TE 160 | Axial T2 HASTE | Axial T1 Dixon | Axial DWI (b50, 400 and 800) |
| --- | --- | --- | --- | --- |
| Field of view | 380 | 380 | 380 | 380 |
| TR (ms) | 1500 | 1200 | 3.97 | 5800 |
| TE (ms) | 104 | 81 | 1.29, 2.52 | 52 |
| Matrix | 320 x 240 | 320 x 260 | 320 x 240 | 134 x 134 |
| Slice/Gap | 3mm at 30% gap | 3.5mm at 20% gap | 3mm at 20% gap | 5mm at 20% gap |
